# Extracellular sombrero vesicles are hallmarks of eosinophilic cytolytic degranulation in tissue sites of human diseases

**DOI:** 10.1093/jleuko/qiae079

**Published:** 2024-03-25

**Authors:** Vitor H Neves, Cinthia Palazzi, Kássia K Malta, Kennedy Bonjour, Felipe Kneip, Felipe F Dias, Josiane S Neves, Peter F Weller, Rossana C N Melo

**Affiliations:** Laboratory of Cellular Biology, Department of Biology, ICB, Federal University of Juiz de Fora, UFJF, Rua José Lourenço Kelmer, Juiz de Fora, MG 36036-900, Brazil; Laboratory of Cellular Biology, Department of Biology, ICB, Federal University of Juiz de Fora, UFJF, Rua José Lourenço Kelmer, Juiz de Fora, MG 36036-900, Brazil; Laboratory of Cellular Biology, Department of Biology, ICB, Federal University of Juiz de Fora, UFJF, Rua José Lourenço Kelmer, Juiz de Fora, MG 36036-900, Brazil; Laboratory of Cellular Biology, Department of Biology, ICB, Federal University of Juiz de Fora, UFJF, Rua José Lourenço Kelmer, Juiz de Fora, MG 36036-900, Brazil; Unity of Biochemistry Membrane and Transport, Department of Cellular Biology and Infection, Institut Pasteur, 75724 Paris Cedex 15, Paris, France; Laboratory of Cellular Biology, Department of Biology, ICB, Federal University of Juiz de Fora, UFJF, Rua José Lourenço Kelmer, Juiz de Fora, MG 36036-900, Brazil; Laboratory of Cellular Biology, Department of Biological Sciences, State University of Minas Gerais, UEMG, Avenida São Paulo 3996, Campus Ibirité, MG 32400-000, Brazil; Institute of Biomedical Sciences, Federal University of Rio de Janeiro, UFRJ, Avenida Carlos Chagas Filho 373, Rio de Janeiro, RJ 21941-971, Brazil; Department of Medicine, Beth Israel Deaconess Medical Center, Harvard Medical School, 330 Brookline Avenue, CLS 943, Boston, MA 02215, United States; Laboratory of Cellular Biology, Department of Biology, ICB, Federal University of Juiz de Fora, UFJF, Rua José Lourenço Kelmer, Juiz de Fora, MG 36036-900, Brazil

**Keywords:** cytolysis, eosinophil activation, eosinophilic diseases, eosinophil sombrero vesicles, ETosis, extracellular vesicles, inflammation, transmission electron microscopy, ultrastructure

## Abstract

Eosinophil sombrero vesicles are large tubular carriers resident in the cytoplasm of human eosinophils, identifiable by transmission electron microscopy, and important for immune mediator transport. Increased formation of sombrero vesicles occurs in activated eosinophils in vitro and in vivo. In tissue sites of eosinophilic cytolytic inflammation, extracellular eosinophil sombrero vesicles are noted, but their frequency and significance in eosinophil-associated diseases remain unclear. Here, we performed comprehensive quantitative transmission electron microscopy analyses and electron tomography to investigate the numbers, density, integrity, and 3-dimensional structure of eosinophil sombrero vesicles in different biopsy tissues from 5 prototypic eosinophil-associated diseases (eosinophilic chronic rhinosinusitis/nasal sinuses, ulcerative colitis/intestines, hypereosinophilic syndrome/skin, dermatitis/skin, and schistosomiasis/rectum). The morphology of extracellular eosinophil sombrero vesicles was also compared with that of cytoplasmic eosinophil sombrero vesicles, isolated by subcellular fractionation from peripheral blood eosinophils. We demonstrated that (i) eosinophil cytolysis, releasing intact sombrero vesicles and membrane-bound granules, is a consistent event in all eosinophil-associated diseases; (ii) eosinophil sombrero vesicles persist intact even after complete disintegration of all cell organelles, except granules (late cytolysis); (iii) the eosinophil sombrero vesicle population, composed of elongated, curved, and typical sombreros, and the eosinophil sombrero vesicle 3-dimensional architecture, diameter, and density remain unchanged in the extracellular matrix; (iv) free eosinophil sombrero vesicles closely associate with extracellular granules; and (v) free eosinophil sombrero vesicles also associate with externalized chromatin during eosinophil ETosis. Remarkably, eosinophil sombrero vesicles appeared on the surface of other cells, such as plasma cells. Thus, eosinophil cytolysis/ETosis can secrete intact sombrero vesicles, alongside granules, in inflamed tissues of eosinophil-associated diseases, potentially serving as propagators of eosinophil immune responses after cell death.

## Teaser

These findings demonstrate that sombrero vesicles are consistently released in vivo from cytolytic eosinophils and persist as extracellular vesicles in inflamed tissues of eosinophilic diseases.

## 1. Introduction

Eosinophils, multifunctional cells of the innate immune system, are notable not only by their assortment of granule-stored cationic proteins, cytokines, and other immune molecules but also by their unique morphology (reviewed in^1–3^). While the highly acidophilic nature of their secretory granules (termed *specific granules*) is classically used to recognize eosinophils in body fluids and tissues by light microscopy,^4^ the ultrastructure of these cells enables their unambiguous identification at high resolution.^5^ This is because eosinophil granules contain in their center a dense crystalloid material embedded in a less dense matrix, features found solely in eosinophils when imaged under transmission electron microscopy (TEM). The simple finding of eosinophil granules in the cytoplasm canonically defines the eosinophil lineage in multiple species, and when found extracellularly deposited in inflammatory sites through cytolytic degranulation, these free extracellular granules (FEGs) act as eosinophil markers (reviewed in^5,6^).

The eosinophil architecture is so unique that its cytoplasm accommodates a very active vesiculotubular system with a particular morphology, promptly identified by TEM, named eosinophil sombrero vesicles (EoSVs). These vesicles, commonly referred to as “sombreros,” constitute a population of large tubular carriers resident in the cytoplasm of human eosinophils (reviewed in^6–8^). EoSVs have a remarkable ability to interact with and bud from secretory granules and, for this reason, are frequently seen around or in contact with these organelles. Our prior investigations have elucidated that the rationale behind such interaction arises from the marked association of EoSVs with the intracellular transport of granule-derived immune mediators (reviewed in^6–9^). Immunoelectron microscopy has detected several immune mediators on EoSVs, including cytokines and CD63, a recognized marker of a granule-delimiting membrane.^8,10,11^

Amplified formation of EoSVs is directly associated with eosinophil activation. Not only the total number of EoSVs distributed throughout the cytoplasm but also the number of EoSVs in interaction with emptying granules increases in response to several stimuli.^10,12^ Accordingly, the numbers of EoSVs are significantly augmented in vivo within hypereosinophilic syndrome (HES) blood eosinophils, which are naturally activated cells compared to healthy donors.^13^ Moreover, robust pools of cytoplasmic EoSVs have been reported in tissue eosinophils during other eosinophil-associated diseases (EADs) such as eosinophilic granulomatosis with polyangiitis^14^ and tumor-associated tissue eosinophilia.^15^

Eosinophil cytolysis with the release of membrane-bound FEGs is a well-documented process of eosinophil secretion observed in a multitude of EADs (reviewed in^2^). In tissue sites of eosinophilic inflammation depicting eosinophil cytolysis, findings of extracellular EoSVs have been attracting attention.^16–18^ However, it remains undefined whether the presence of cell-free EoSVs is a frequent event in vivo as observed for FEGs, as well as the potential functional roles of these structures. Here, we performed comprehensive TEM analyses to investigate at high resolution the occurrence, distribution, integrity, and 3-dimensional (3D) structure of EoSVs in different tissues of human biopsies of prototypical EADs. Our findings provide evidence that intact EoSVs are consistently released from cytolytic eosinophils, persist as extracellular vesicles in inflamed tissues after cell death, and potentially interact with other cells.

## 2. Materials and methods

### 2.1 Ethics statement

Eosinophils isolated from the peripheral blood and biopsy samples were obtained from the Beth Israel Deaconess Medical Center (BIDMC) in accordance with the ethical principles taken from the Declaration of Helsinki, and written informed consent was obtained from donors. Institutional review board (IRB) approval was obtained annually from the BIDMC Committee on Clinical Investigation (Boston, MA, USA), with the most recent IRB approved protocol number 2001P000561. For schistosomiasis mansoni (SM) biopsies, kindly provided by Dr. Aloísio Sales da Cunha, all medical and surgical procedures were performed at the Alfa Institute of Gastroenterology of the University Hospital (Hospital das Clínicas) at Federal University of Minas Gerais (UFMG), as approved by the Ethics Committee of this institution (Protocol ETIC 204/06), with consent from all patients.

### 2.2 Eosinophil isolation and subcellular fractionation

Eosinophils were isolated from the peripheral blood of healthy donors as described^19^ and purified by negative selection using human eosinophil-enrichment cocktail (StemSep; StemCell Technologies) and the magnetic antibody cell-sorting (MACS) bead procedure (Miltenyi Biotec). Eosinophil purity was >99%. For subcellular fractionation,^20^ eosinophils (10 to 30 × 10^6^) were resuspended in disrupting buffer supplemented with 5 mg/mL 1,4-dithio-DL-threitol (DTT) and subjected to nitrogen cavitation (Parr) (600 psi, 10 min). Postnuclear supernatants, recovered after centrifugation (400 × g, 10 min), were ultracentrifuged (100,000 × g, 1 h at 4 °C) in linear isotonic Optiprep (Axis-Shield) gradients (0% to 45% in disrupting buffer without protease inhibitors). The vesicle-rich fraction of eosinophils was then resuspended in molten 2% agar in 0.1 M sodium cacodylate buffer, pH 7.4, and quickly recentrifuged.^13^ Resultant agar pellets were kept in the same buffer at 4 °C for further processing.

### 2.3 Biopsy samples

Tissue biopsy samples were obtained from patients with varied inflammatory diseases/conditions. The specimens were obtained from (i) the ileum, rectum, or continent pouches of 16 patients with ulcerative colitis (UC) with traditional surgical pathology criteria of the resected specimens used for diagnosis; (ii) the nasal sinuses of 2 patients with eosinophilic chronic rhinosinusitis (ECRS); (iii) skin lesions of 2 patients with hypereosinophilic syndrome (HES) negative for FIP1-like 1/platelet-derived growth factor α mutation; (iv) skin from 3 patients with acute inflammation associated with the recruitment of granulocytes (stem cell factor–induced dermatitis) as previously described;^21^ and (v) rectal diverticulum from 1 patient diagnosed with the pseudo-tumoral form of SM.

### 2.4 TEM

Tissue fragments and EoSV-containing fractions from subcellular fractionation were prepared for TEM using protocols established by our group.^19,22^ Samples were fixed using a mixture of freshly prepared aldehydes [final concentration of 1% paraformaldehyde and 2.5% glutaraldehyde (EM grade, 50% aqueous; Electron Microscopy Sciences–EMS)] in 0.1 M sodium cacodylate buffer, pH 7.4, for 4 h at room temperature (RT). After washing with 0.05 M sodium maleate buffer, pH 5.2, samples were stained en bloc in 2% uranyl acetate in 0.05 M sodium maleate buffer, pH 6.0, for 2 h at RT and washed in the same buffer as before prior to dehydration in graded ethanols and infiltration and embedding with a propylene oxide–Epon sequence (Eponate 12 Resin; Ted Pella). Additional samples were postfixed in 2% aqueous osmium tetroxide and 1.5% potassium ferrocyanide in 0.1 M sodium phosphate buffer, pH 6.0 (reduced osmium), before dehydration and embedding as above. After polymerization at 60 °C for 16 h, thin sections were cut using a diamond knife on an ultramicrotome (Leica). Sections were mounted on uncoated 200-mesh copper grids (Ted Pella) before staining with lead citrate and viewed with a transmission electron microscope (CM 10; Philips or Tecnai G2-20-ThermoFischer Scientific/FEI 2006) at 80 to 120 KV.

### 2.5 Quantitative TEM analyses

Electron micrographs showing inflamed tissues with infiltrated eosinophils were randomly acquired at different magnifications (total *n* = 907 electron micrographs), and a total of 166,900 µm^2^ of tissue area of biopsy samples was analyzed (83,800 µm^2^ for UC, 30,700 µm^2^ for ECRS, 30,500 µm^2^ for HES skin, 11,100 µm^2^ for dermatitis, and 10,800 µm^2^ for SM). To investigate the processes of eosinophil secretion, all eosinophils found in the tissue areas, including regions with FEGs, were scored. Secretory processes were identified and quantitated as piecemeal degranulation (PMD); classical/compound exocytosis or cytolysis as described^5,19,23^ and the percentages of these processes were established.

To evaluate the distribution of free EoSVs in the extracellular matrix and association with FEGs, the distances between EoSVs and FEGs in µm were measured and the proportions of EoSVs were established as follows: <1 µm, >1 µm, or in contact. In total, 3,805 free EoSVs were counted (1,050 for ECRS, 1,771 for UC, 486 for HES, 330 for dermatitis, and 168 for SM). When ETs were present in the tissue inflammatory sites, the distances between EoSVs and ETs were also established.

To assess the numbers and density of EoSVs, randomly found in inflammatory sites, eosinophils were categorized into 3 groups: (i) intact, showing the entire cell profile with undamaged plasma membrane; (ii) in early cytolysis, with a partial rupture of the plasma membrane (up to 30% of plasma membrane loss) and minimal externalization of cytoplasmic contents; and (iii) late cytolysis, showing extensive disruption or total loss of the plasma membrane integrity, total extravasation or loss of cytoplasmic contents, and deposition of membrane-bound secretory granules in the extracellular matrix. Quantitative analyses were performed in electron micrographs from 2 prevalent diseases (ECRS and UC)^24,25^ characterized by a higher rate of eosinophil cytolysis in target organs (nasal sinus and intestine, 54% and 64%, respectively, as established above). Electron micrographs were obtained at the same magnification (6,800×), with each micrograph corresponding to a tissue area of 180 µm². Electron micrographs showing small-sized areas of late cytolysis with a minimal number of FEGs (<5 granules) were disregarded for these analyses.

A total of 238 electron micrographs were analyzed (160 for UC and 78 for ECRS) and the numbers and density (EoSVs/μm²) of EoSVs were established in association with intact or cytolytic (early or late cytolysis) eosinophils. A total of 6,026 EoSVs were counted (*n* = 3,884 for UC and 2,142 for ECRS). Subsequently, the diameter of free EoSVs with typical sombrero morphology was measured and compared among the 3 groups of scored eosinophils (intact or in early or late cytolysis). For each of these groups, 30 EoSVs were randomly selected for diameter evaluation in ECRS and UC samples, totalizing 90 vesicles/disease.

All quantitative analyses were performed using Fiji ImageJ software (National Institutes of Health).

### 2.6 Electron tomography

Electron tomograms, reconstruction, and segmentation were performed as in previous works from our group.^26,27^ After EM processing, serial sections 200 nm thick were collected on 100 mesh-formvar–coated grids. Samples were contrasted with lead citrate and then tilt series were acquired at 120 kV on a Tecnai Spirit G12 microscope (Thermo Fisher Scientific/FEI) with an Eagle 4 k × 4 k camera (Thermo Fisher Scientific/FEI). Tomograms were generated using the SerialEM software^28^ at a magnification of 23,000×. Images in a tilt series were collected at intervals of −65 to +65 in 1° intervals by 1-s exposure per micrograph. All tilted images were aligned and binned by 2 into 1 k × 1 k using the IMOD software package, and 3D reconstructions were calculated using the weighted back-projection. A total of 7 tomograms were analyzed to characterize free EoSV morphology. Modeling was carried out using drawing tools with interpolation in the IMOD software.^29,30^

### 2.7 Statistical analyses

The results are presented as percentages or mean ± SEM, and significance was considered as *P* < 0.05. Data on EoSV populational density (integrity quantification) were analyzed with the nonparametric Kruskal–Wallis test and Dunn's multiple comparison test, and EoSV diameters were analyzed with 1-way analysis of variance and Tukey's multiple comparison test. All analyses were performed using the software GraphPad Prism version 7.00 (GraphPad Software).

## 3. Results

### 3.1 Extracellular sombrero vesicles are a consistent finding associated with eosinophilic cytolytic inflammation

**Fig. 1.**
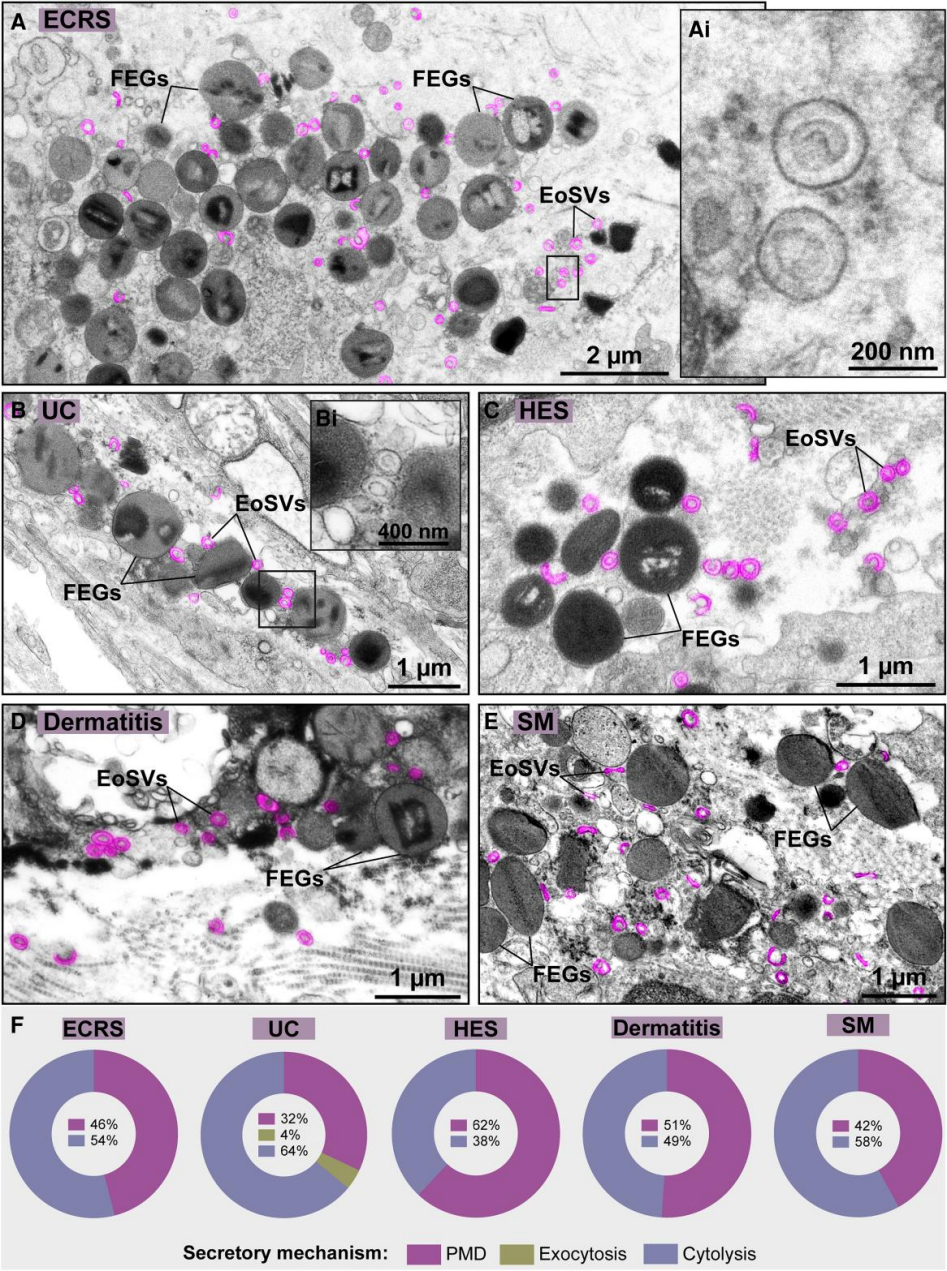
Extracellular sombrero vesicles are frequently observed in tissue sites of eosinophilic cytolytic inflammation. (A–E) Representative electron micrographs of (A) ECRS (nasal sinus), (B) UC (intestines), (C) HES (skin), (D) dermatitis (skin), and (E) SM (rectum) show deposition of FEGs and EoSVs (colored in pink) in the inflamed tissues. Boxed areas in (Ai and Bi) show higher magnifications of EoSVs depicting typical morphology. (F) Quantitative analyses of 166,900 µm^2^ of tissue area of biopsy samples demonstrated the predominance or high incidence of cytolysis in the eosinophil-associated conditions. Samples were prepared for TEM.

The presence of free EoSVs in the extracellular matrix has been noticed in tissue sites associated with eosinophilic inflammation.^16–18^ To better understand whether the occurrence of extracellular EoSVs is a frequent event in vivo, we performed comprehensive TEM analyses in vast areas of tissue biopsies from patients with EADs/inflammation. Five different conditions, including a parasitic disease, all of them characterized by recruitment and activation of eosinophils (ECRS, UC, HES, dermatitis, and SM), were evaluated (Supplementary Table 1). By analyzing a total of 166,900 μm^2^ of target tissues (Supplementary Table 1), we found 3,805 extracellular EoSVs. Remarkably, these vesicles were seen in all tissue inflammatory sites with evidence of eosinophilic cytolytic degranulation (Fig. 1A–E), a process found at high frequency (38% to 64%) in all disorders studied here (Fig. 1F).

### 3.2 Free EoSVs remain in inflamed tissues after cell death

**Fig. 2.**
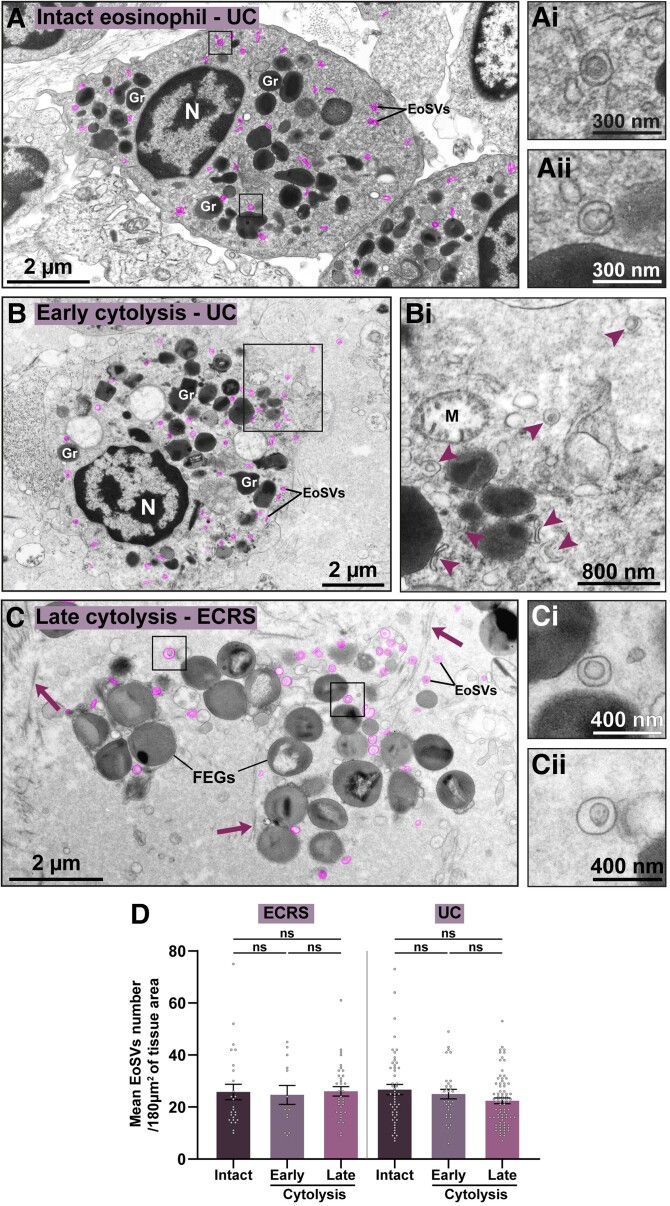
Free EoSVs persist in inflamed tissues after eosinophil cytolysis. (A–C) Representative electron micrographs showing eosinophils as intact (A) or in process of early (B) or late (C) cytolysis, as described in Materials and methods. The intact eosinophil shows secretory granules (Gr) with evidence of piecemeal degranulation (granule enlargement, core disarrangement, and mobilization).^50^ Cytoplasmic EoSVs (colored in pink) with typical morphology are shown (boxes) in higher magnification in (Ai and Aii). (B) Early cytolytic eosinophil displaying partial plasma membrane breakup (boxed area). Cytoplasmic and released forms of EoSVs are indicated (arrowheads) in (Bi). Note a collapsing mitochondrion (M). (C) Membrane-bound FEGs and free EoSVs (pink, boxed areas in Ci and Cii) are seen in the extracellular matrix after the complete disintegration of the plasma membrane and other organelles. Arrows indicate collagen fibrils. (D) The density of the EoSV population did not change when associated with intact or cytolytic eosinophils. Quantitative analyses were performed in biopsy samples from ECRS (nasal sinus) and UC (intestine) prepared for TEM. A total of 238 tissue areas (each of them with 180 μm^2^) were analyzed (78 from ECRS and 160 from UC). A total of 6,026 EoSVs were counted (*n* = 2,142 for ECRS and *n* = 3,884 for UC). Results are expressed as mean ± SEM (ns, not significant, *P* > 0.05). N, nucleus.

Since extracellular EoSVs were associated with eosinophil cytolysis, we next interrogated whether these vesicles remained intact in the extracellular matrix following cell death and organellar disintegration. To answer this question, we chose 2 prevalent diseases (ECRS and UC)^24,25^ characterized by a higher rate of eosinophil cytolysis in target organs (nasal sinus and intestine, 54% and 64%, respectively) (Fig. 1F)^18^ and categorized all eosinophils randomly found in inflammatory sites as follows: (i) intact (showing the entire cell profile with intact plasma membrane) (Fig. 2A), (ii) in the process of early cytolysis (showing partially disrupted plasma membrane—up to 30% of plasma membrane loss) (Fig. 2B), and (iii) in the process of late cytolysis (showing extensive disruption or total loss of the plasma membrane integrity and presence of FEGs) (Fig. 2C). The numbers and density of EoSVs were then established in association with all 3 situations (total *n* = 6,026 sombreros).

Our findings revealed that the mean density of EoSVs (EoSVs/180 µm² of tissue area) remained remarkably consistent across all 3 groups, for both diseases (Fig. 2D). These EoSVs values associated with eosinophils as intact cells or undergoing early or late cytolysis were, respectively, as follows (mean ± SEM): 27.5 ± 3.4, 24.7 ± 3.6, and 28.4 ±2.3 for ECRS (*n* = 2,142) (*P* > 0.05) and 26.7 ± 1.9, 25 ± 1.7, and 22.4 ± 1.1 for UC (*n* = 3,884) (*P* > 0.05). Intact eosinophils showed clear signs of activation with mobilized granules with signs of PMD (Fig. 2A), a secretory process commonly found in addition to cytolysis (Fig. 1F). Interestingly, when intact and disrupted eosinophils were imaged in the same inflammatory tissue site, intracellular and extracellular EoSVs could be compared side by side, and they exhibited apparently identical morphologies (Supplementary Fig. 1).

Altogether, our findings demonstrate that not only the population of secretory granules but also the population of EoSVs are preserved after cell death.

### 3.3 Morphological characterization of extracellular EoSVs

**Fig. 3.**
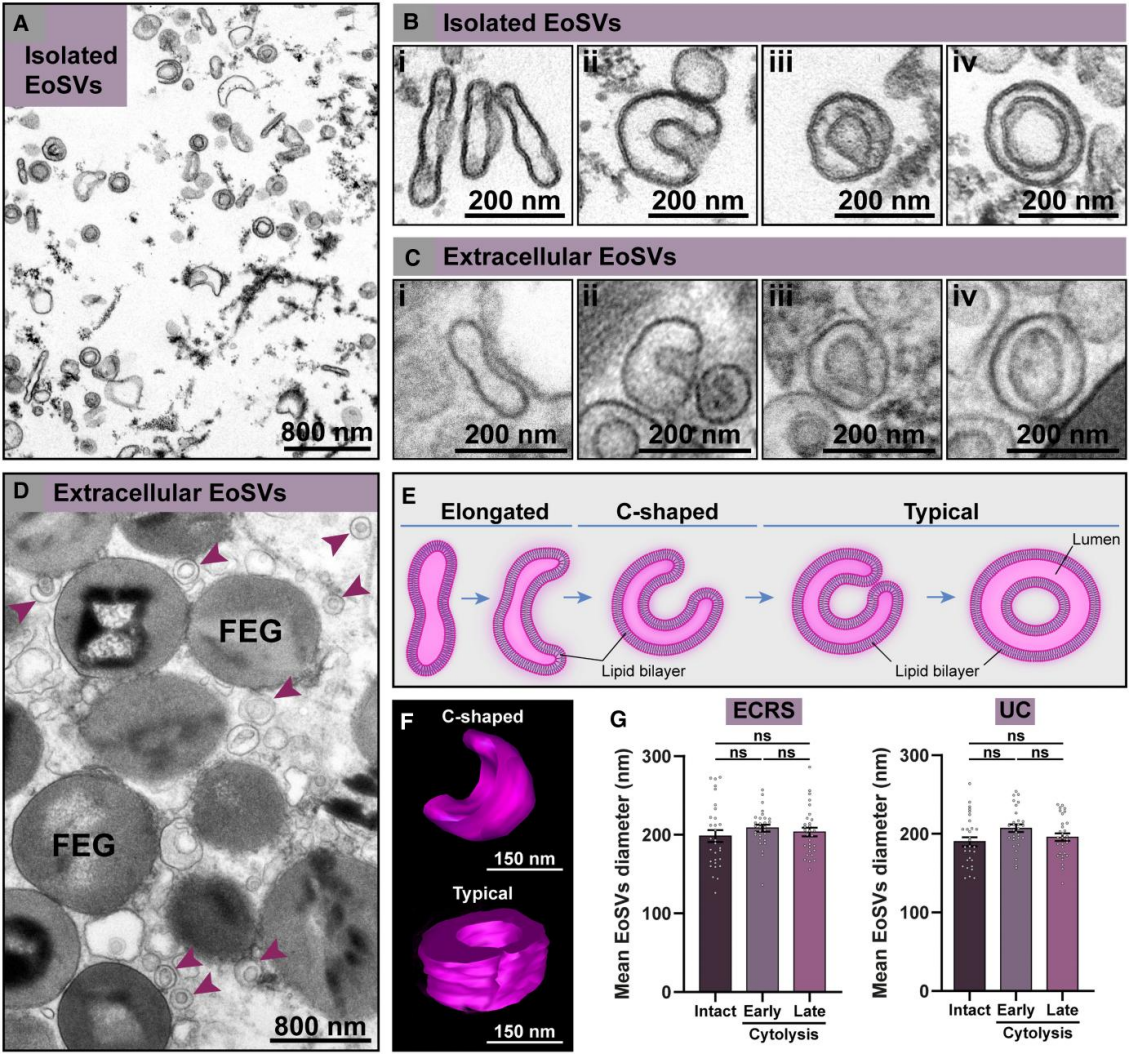
Morphological characterization of cell-free sombrero vesicles (EoSVs). (A, B) A fraction of isolated EoSVs by subcellular fractionation from peripheral blood human eosinophils. (B) Higher magnification of isolated EoSVs showing the morphology and the trilaminar aspect of the vesicles membranes. (C, D) Extracellular tissue EoSVs showing intact membranes and the same morphologies as seen in (B). Both cytoplasmic and extracellular EoSVs appear as elongated (Bi, Ci), curved with varying curvatures, including C-shaped (Bii, Cii), and round (typical sombreros) showing (Biii, Ciii) or not (Biv, Civ) the contacted opposing tubular edges. These morphologies are illustrated in (E) and shown in 3 dimensions by electron tomography in (F). Reconstructions were done from an extracellular C-shaped EoSV present in inflamed tissue and a typical cytoplasmic EoSV. (G) Quantitative analysis revealed that the mean diameter of EoSVs with typical sombrero morphology did not change when comparing vesicles from intact cells and late and early cytolytic eosinophils in both ECRS and UC. A total of 180 EoSVs (90 for each condition) were randomly selected and analyzed. Samples from isolated EoSVs and biopsies from patients with ECRS (*n* = 2) and UC (*n* = 16) were prepared for TEM. Results are expressed as mean ± SEM (ns, not significant, *P* > 0.05). FEG, free extracellular granule. See also Supplementary Movie 1 (Supplementary material).

Subsequently, we sought to ascertain whether cell-free EoSVs had the same morphology as seen in the cytoplasm of human eosinophils. To address this question, we analyzed in detail the morphology of individual EoSVs. First, EoSVs were isolated from the cytoplasm of peripheral blood eosinophils using subcellular fractionation after negative selection.^12,20^ As previously demonstrated, EoSVs can be isolated as a distinct population localized in fractions slightly less dense than granule-containing fractions.^12^ EoSVs found in this fraction (Fig. 3A) appeared as vesiculotubular structures composed of elongated (Fig. 3Bi) and curved tubules with an array of curvatures ranging from slightly to highly curved (C-shaped) (Fig. 3Bii) until complete apposition of the 2 tubule edges. When the 2 edges of the tubule were connected or apparently fused, the typical sombrero appearance was visualized as a structure with double membranes (Fig. 3Biii and Biv). At high magnification, the trilaminar aspect of these membranes, a feature of cellular membranes when imaged by TEM,^5^ was feasibly identified (Fig. 3Bi–Biv). Quantitative analyses of a total of 75 vesiculotubular structures randomly found in the isolated fraction showed that 33% of the EoSV population was composed of elongated or slightly curved sombrero vesicles (Fig. 3Bi), 7% were C-shaped vesicles (Fig. 3Bii), and 60% had typical sombrero morphologies (Fig. 3Biii and Biv).

Second, we evaluated single EoSVs free in the extracellular matrix in tissue sites in which only FEGs were present (Fig. 3C and D). We found the same population and morphology of EoSVs as depicted in the cytoplasm: elongated (Fig. 3Ci), C-shaped (Fig. 3Cii), and typical sombreros (Fig. 3Ciii and Civ). The trilaminar feature of the surrounding double membrane was clearly identified, thus demonstrating that these vesicles were preserved, even after complete cell death and the disappearance of all other organelles, except the secretory granules (Figs. 1Ai and 3Ci–Civ, D). Application of electron tomography in these tissue sites showed in 3 dimensions the architecture and spatial distribution of extracellular EoSVs (Supplementary Movie 1), thus confirming their preservation outside the cell. In accord with our previous studies (reviewed in^6,7^), it is clear that the population of EoSVs, both cytoplasmic and extracellular, is composed of highly plastic vesiculotubular structures, able to bend and exhibiting large surface areas (Fig. 3E), as highlighted from our 3D electron tomographic analyses (Fig. 3F).

Next, we questioned if the diameter of EoSVs would change during the cytolysis process and when free in the extracellular medium. Our quantitative analyses of EoSVs from ECRS and UC biopsies showed that the mean diameter of typical sombreros did not differ during cytolysis compared to cytoplasmic sombreros evaluated in intact cells. The diameter (nm) of EoSVs from intact, early, and late cytolytic eosinophils, respectively, were as follows (mean ± SEM): 198.4 ± 7.5, 208.6 ± 4.4, and 203.5 ± 5.5 for ECRS (*n* = 90 sombreros) (*P* > 0.05) and 193 ± 5.9, 210.4 ± 4.9, and 199 ± 4.9 for UC (*n* = 90 sombreros) (*P* > 0.05) (Fig. 3G).

Altogether, our findings demonstrated that the density, architecture, and diameter of EoSVs did not change even after complete cell disintegration.

### 3.4 Extracellular EoSVs are closely associated with FEGs and eosinophil extracellular traps

**Fig. 4.**
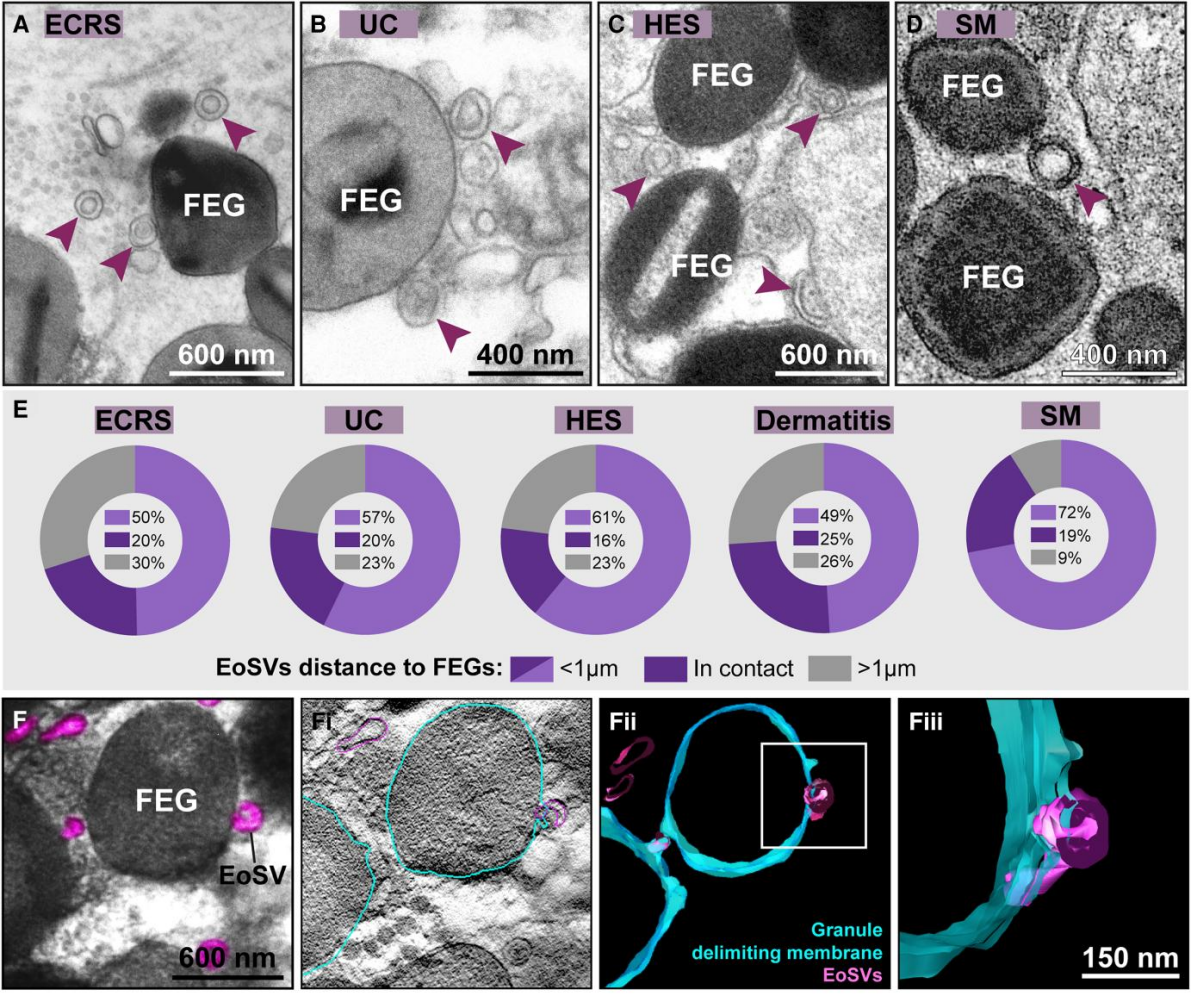
Extracellular sombrero vesicles associate with FEGs in inflamed tissues. (A–D) Representative electron micrographs showing intact extracellular EoSVs (arrowheads) around or in contact with FEGs in different inflamed tissues of eosinophilic diseases. (E) Quantitative TEM analyses of tissue sites with eosinophils in late cytolysis demonstrate that most free EoSVs (70% to 91%) in all diseases were found close to (<1 µm of distance) or in direct contact with FEGs while 9% to 30% were localized at distances higher than 1 µm from FEGs. A total of 3,805 EoSVs (1,050 for ECRS, 1,771 for UC, 486 for HES, 330 for dermatitis, and 168 for SM) were counted and the distances for EoSVs–FEGs established. (F) Electron micrograph showing FEGs and extracellular sombreros (pink) found in an inflamed skin (dermatitis). (Fi) A representative virtual slice extracted from the tomogram acquired from (F) was partially traced in blue (granule-delimiting membranes) and pink (EoSV membranes) to generate 3D models as seen in (Fii and Fiii). Note that a sombrero vesicle is fused with the granule membrane [boxed area seen in higher magnification in (Fiii)]. Biopsy samples from ECRS (nasal sinus), UC (intestine), HES (skin), SM (rectum), and dermatitis were prepared for TEM.

In the eosinophil cytoplasm, particularly within activated eosinophils, EoSVs are frequently seen in contact or around specific granules (reviewed in^6,7^). Our analyses of tissue biopsies strongly indicated that this relationship is maintained extracellularly (Fig. 1A–E and Fig. 4A–D). To get more insights into this event, we next evaluated the FEG–EoSV association in more detail using both quantitative TEM and 3D electron tomography. We found that most free EoSVs were situated nearby (<1 µm of distance) to FEGs (Fig. 4E). This proximity was strikingly evident across all conditions, reaching 70% for ECRS, 77% for UC and HES, 74% for dermatitis, and 91% for SM (Fig. 4E). Intriguingly, part of the EoSV population was in direct (physical) contact with FEGs (accounting for 20% in ECRS and UC, 16% in HES, 25% in dermatitis, and 19% in SM) (Fig. 4E). Electron tomographic reconstructions revealed that these contact sites represent fusion areas between granules and sombreros (Fig. 4F and 4Fi–Fiii).

**Fig. 5.**
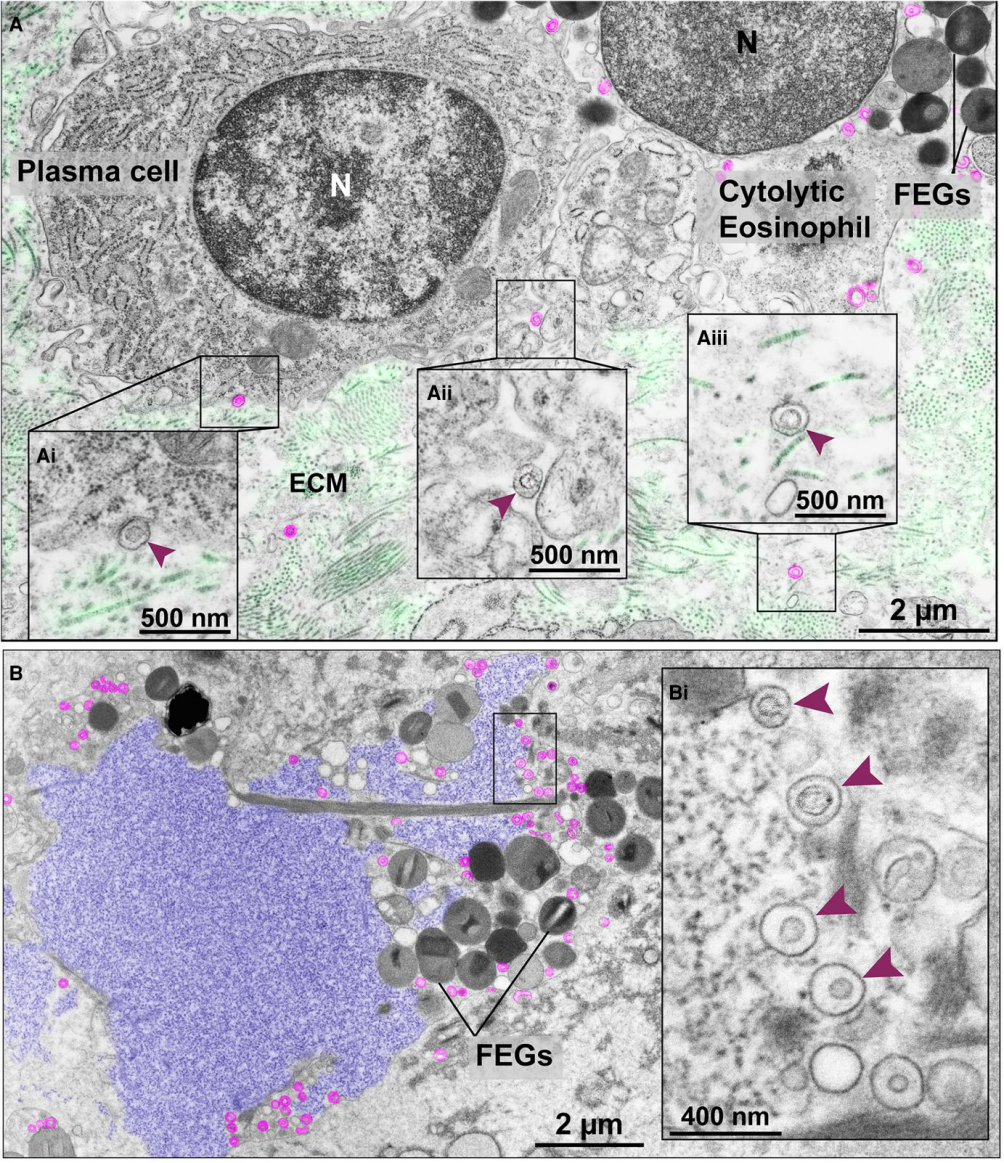
Extracellular EoSVs in tissue sites of cytolytic eosinophilic inflammation. (A) Intact EoSVs (colored in pink) are seen around a cytolytic eosinophil with FEGs, on the surface (Ai and Aii, arrowheads) of a plasma cell and dispersed in the extracellular matrix (ECM) (Aiii, arrowhead). (Ai–Aiii) are higher magnifications of the boxed areas. Longitudinal and cross sections of collagen fibrils from the ECM were colored in light green. In (B), a typical late EETosis area showing highly decondensed and released chromatin (colored in purple), which is the appearance of ETs as seen in thin sections.^18^ Note intermingled FEGs and intact extracellular sombreros (pink) seen in higher magnification in (Bi) (arrowheads). The electron micrographs were taken from ulcerative colitis/intestine (A) and ECRS/nasal sinus (B) biopsy samples prepared for TEM. N, nucleus.

While most free EoSVs were seen close to FEGs (Fig. 4E), our results also demonstrate that free intact EoSVs can be found at distances greater than 1 μm from granules in all diseases investigated (Fig. 4E) and reach long distances in the extracellular matrix (up to 12 μm). This is particularly interesting because EoSVs as transport carriers of granule-stored products (reviewed in^8^) may have the potential to interact with other cells. Indeed, our comprehensive analyses of massive areas of tissues showed evidence in this direction with the observation of EoSVs on the surface of other cells, such as plasma cells (Fig. 5A).

In sites of eosinophilic inflammation containing FEGs and at times Charcot–Leyden crystals (CLCs), free EoSVs were also frequently seen close to and/or intermingled with extracellular, expanded, and highly decondensed chromatin. This is an ultrastructural feature of the late stage of eosinophil ETosis (EETosis), i.e. chromatin released as extracellular traps (ETs), as revealed in thin sections by TEM.^18^ In these tissue sites, 23% to 45% of EoSVs were close (<1 μm distance) or completely immersed in the externalized chromatin (Fig. 5B and Bi).

## 4. Discussion

In recent decades, the phenomenon of eosinophil cytolysis with generation of FEGs, observed in vivo in inflamed tissues for more than a century, has garnered considerable attention. Many studies, especially from the group of Carl Persson and Jonas Erjefält, provided evidence that eosinophil cytolysis represented an “ultimate activation” of this cell in tissues and secretions, and the occurrence of this process and resulting release of FEGs constituted an important mechanism of eosinophil degranulation (e.g.^23,31–41^). This new paradigm of eosinophil activation and secretion taking shape during those years is currently considered a remarkable feature of the eosinophil biology (reviewed in^2^).

Here, we demonstrate that, in addition to FEGs, intact EoSVs are also deposited in tissue sites of eosinophilic cytolytic inflammation and persist in the extracellular medium as a result of eosinophil secretion. By performing comprehensive ultrastructural quantitative analyses and 3D electron tomography using biopsies from 5 prototypic EADs, we found that (i) cytolysis is a common process of eosinophil degranulation, in accord with previous studies as mentioned above; (ii) eosinophil cytolysis leads to the release of EoSVs together with secretory granules; (iii) EoSVs remain intact in the extracellular matrix even after complete disintegration of all cell organelles, except granules (late cytolysis); (iv) the typical architecture of free EoSVs did not change when in the extracellular matrix compared to cytoplasmic EoSVs; (v) free EoSVs intermingle with decondensed externalized chromatin in tissue sites of EETosis; and (vi) EoSVs are associated with other immune cells, suggesting a potential role for these extracellular vesicles in intercellular communication.

When seen under TEM, biological membranes show a characteristic “trilaminar” appearance (2 dense outer lines and a less dense inner region). This is because osmium, the heavy metal used during EM preparation, binds preferentially to the polar head groups of the lipid bilayer, thus producing the trilaminar pattern.^5^ TEM was extensively used in the past, and it is still a gold standard technique to reveal that clusters of eosinophil granules are kept intact in vivo, allowing not only their identification but also the visualization of their preserved delimiting membranes (membrane-bound organelles) once in the extracellular matrix.^5^ Our present work using both conventional and 3D TEM consistently demonstrate that the EoSV membranes (Figs. 1Ai and 3Ci–Civ, 5Bi), architecture (Supplementary Movie 1), and numbers (Fig. 2D) are well maintained extracellularly in vivo after cell death.

One interesting aspect detected by our analyses in inflammatory tissue sites is the striking association between free EoSVs and FEGs. Most (70% to 91%) extracellular EoSVs (*n* = 3,085) were in contact or distributed less than 1 µm of distance from FEGs (Fig. 4E). This fact may be reflecting the prominent intracellular EoSV–granule relationship as unveiled by our previous studies demonstrating that eosinophil activation induces the production of cytoplasmic EoSVs, increases their interaction with and budding from granules, and leads to newly formed EoSV redistribution in the peripheral cytoplasm.^10–13^ Cytolytic eosinophils are highly activated cells, as pointed out by earlier works,^31–37,40^ and numerous EoSVs in tissue sites of cytolytic inflammation, here identified, substantiate this concept. Eosinophils in the process of cytolysis are producing EoSVs from granules while in the cytoplasm and likely in the extracellular matrix. Our tomographic analyses captured images showing EoSVs fused to FEGs (Fig. 4Fi–Fiii), which indicates that granules, as ligand-responsive, secretion-competent organelles outside the cell,^42^ may be releasing EoSVs. However, additional studies are needed to prove that EoSVs can be generated in situ from FEGs.

More recently, cytolysis has gained further discussion with the discovery of EETosis, meaning eosinophil cytolysis associated with the release of ETs and the formation of CLCs.^43–46^ Our present study shows that in sites in which EETosis is occurring, FEGs and free EoSVs are seen intermingled with released chromatin, corroborating a previous study from our group.^18^ This means that EETosis-mediating granule secretion is also involved in the release of EoSVs (Fig. 5B and Bi).

The findings of robust pools of extracellular EoSVs in all 5 types of EADs investigated (ECRS, UC, HES, dermatitis, and SM) and in different tissues (skin, nasal sinus, and intestines) have the important following implications: (i) secretion of EoSVs through cytolysis is a consistent event in vivo, and (ii) EoSVs may have functional roles outside the cell associated with the traffic of immune mediators and intercellular communication. The steady presence and high number of free EoSVs in sites of eosinophilic cytolytic inflammation are in accord with a previous study evaluating biopsies of 9 patients with eosinophilic esophagitis.^16^ In this study of esophageal specimens, more than 80% of eosinophils recognized by TEM were undergoing cytolysis with numerous sombrero vesicles detected extracellularly.^16^ The occurrence of free EoSVs in cytolytic sites of other diseases such as giardiasis has also been reported.^17^

The discovery of EoSVs as an active vesicular system involved in the intracellular transport of immune mediators was made in 2005 by the Weller group.^12^ Since then, several compounds pertinent to eosinophil functionality have been identified on these vesicles with direct molecular visualization using an optimized immunonanogold technique for precise immunolabeling of membranous compartments in leukocytes.^47^ Cationic proteins (major basic protein 1),^13^ tetraspanins (CD63),^10^ snares (syntaxin 17),^48^ interleukins [IL-4, interferon γ (IFN-γ)],^11,12^ and their cognate receptors (IL-4Rα chain)^9^ have been immunolocalized on EoSVs. Moreover, quantitative TEM enabled the evaluation of immunolabeled EoSVs during immune responses. For example, we found that the numbers of IFN-γ–positive EoSVs per cell section had more than a 200% increase in the cytoplasm in response to cell activation with tumor necrosis factor α. Yet, IFN-γ–immunolabeled EoSVs presented a differential distribution in the cytoplasm, with higher numbers in the peripheral cytoplasm (within 1 μm of the plasma membrane) compared to the adjacent cytoplasmic area deeper in the cell,^11^ thus indicating that these vesicles were mobilized for extracellular release.

Are EoSVs functional outside the cell? As membrane-bound, morphologically intact vesicles, able to pack eosinophil products, free EoSVs can be characterized as extracellular vesicles fully capable of carrying granule-derived cargos at long distances. In support of this, our quantitative analyses detected that 9% to 30% of free EoSVs were distributed with a distance greater than 1 μm from granules, competent to reach long distances in the extracellular matrix (up to 12 μm distance from granules). While we cannot demonstrate here the ability of free EoSVs to transport immune mediators because of the technical limitations regarding the fixative used at the time of biopsy collection, we suggest that free EoSVs are involved in the trafficking of eosinophil immune mediators extracellularly. As vesiculotubular structures with high surface-to-volume ratios, EoSVs are considered advantageous for the accommodation and rapid translocation of membrane-bound proteins and other products derived from secretory granules within the eosinophil cytoplasm.^8^ As extracellular vesicles, the EoSV plasticity and architecture may facilitate intercellular communication. The finding of EoSVs on the surface of plasma cells (Fig. 5A) is indicative that EoSVs may act as messengers among immune cells, an important function of extracellular vesicles in the context of the immune system.^49^ The contribution of EoSVs in this process needs to be addressed in future studies to explore their biological significance as eosinophil extracellular vesicles.

In summary, we show that eosinophil cytolysis, as an important mechanism of eosinophil activation and secretion in vivo, leads to consistent release of EoSVs, which remain intact in the extracellular matrix as free vesicles after cell death. The persistence of EoSVs in tissue sites of eosinophilic cytolytic inflammation, together with membrane-bound specific granules, is a frequent event during EADs and, as potentially functional extracellular vesicles, may be involved in the mediation of eosinophil immune responses.

## Supplementary Material

qiae079_Supplementary_Data

## Abbreviations

CLC: Charcot–Leyden crystal
EAD: eosinophil-associated disease
ECRS: eosinophilic chronic rhinosinusitis
EETosis: eosinophil ETosis
EM: electron microscopy
EoSV: eosinophil sombrero vesicle
ET: extracellular trap
FEG: free extracellular granule
HES: hypereosinophilic syndrome
IFN-γ: interferon γ
IL: interleukin
PMD: piecemeal degranulation
SM: schistosomiasis mansoni
TEM: transmission electron microscopy
UC: ulcerative colitis
