# Supplementary material for: Extracellular sombrero vesicles are hallmarks of eosinophilic cytolytic degranulation in tissue sites of human diseases
Source: J Leukoc Biol. 2024 Mar 25;116(2):398–408. doi: 10.1093/jleuko/qiae079 (PMC11271979; doi:10.1093/jleuko/qiae079)
Supplement: qiae079_Supplementary_Data [file qiae079_supplementary_data.zip › Movie_S1_legend.pdf]

Movie S1 – Electron tomography from an inflamed tissue site (skin, dermatitis), characterized by late eosinophil cytolysis, showing free extracellular granules and intact sombrero vesicles (colored in pink).
