# Supplementary material for: Extracellular sombrero vesicles are hallmarks of eosinophilic cytolytic degranulation in tissue sites of human diseases
Source: J Leukoc Biol. 2024 Mar 25;116(2):398–408. doi: 10.1093/jleuko/qiae079 (PMC11271979; doi:10.1093/jleuko/qiae079)
Supplement: qiae079_Supplementary_Data [file qiae079_supplementary_data.zip › Table_S1.pdf]

EXTRACELLULAR SOMBRERO VESICLES ARE HALLMARKS OF EOSINOPHILIC CYTOLYTIC DEGRANULATION IN TISSUE SITES OF HUMAN DISEASES

Vitor H. Neves[1], Cinthia Palazzi[1], Kássia K. Malta[1], Kennedy Bonjour[1,2], Felipe Kneip[1], Felipe F. Dias[3], Josiane S. Neves[4], Peter F. Weller[5], Rossana C. N. Melo[1*]

[1] Laboratory of Cellular Biology, Department of Biology, ICB, Federal University of Juiz de Fora, UFJF, Rua José Lourenço Kelmer, Juiz de Fora, MG, 36036-900, Brazil
[2] Unity of Biochemistry Membrane and Transport, Department of Cellular Biology and Infection, Institut Pasteur, Paris 75724 Paris Cedex 15, France
[3] Laboratory of Cellular Biology, Department of Biological Sciences, State University of Minas Gerais (UEMG), Campus Ibirité, MG, Brazil
[4] Institute of Biomedical Sciences, Federal University of Rio de Janeiro, UFRJ, Rio de Janeiro, Brazil
[5] Department of Medicine, Beth Israel Deaconess Medical Center, Harvard Medical School, 330 Brookline Avenue, CLS 943, Boston, MA, 02215, USA
*Correspondence: rossana.melo@ufjf.br

Table S1 – Summary of the analyzed samples

**Condition**	**Tissue sample**	**Patients (n)**	**Tissue fragments (n)**	**Tissue area (µm²)**
Ulcerative colitis	Ileum, rectum or continent pouches	16	24	83,800
Eosinophilic chronic rhinosinusitis	Nasal sinus	2	8	30,700
Hypereosinophilic syndrome	Skin	2	5	30,500
Dermatitis	Skin	3	5	11,100
Schistosomiasis mansoni	Rectum	1	3	10,800
